# *MSTN* Regulates Bovine Skeletal Muscle Satellite Cell Differentiation via *PSMA6*-Mediated AKT Signaling Pathway

**DOI:** 10.3390/ijms26114963

**Published:** 2025-05-22

**Authors:** Tengxia Ma, Meiling Miao, Xiangquan Liu, Linlin Zhang, Yiwen Guo, Xin Li, Xiangbin Ding, Hong Guo, Debao Hu

**Affiliations:** Key Laboratory of Animal Breeding and Healthy Livestock Farming, College of Animal Science and Veterinary Medicine, Tianjin Agricultural University, Tianjin 300392, China; 2203010112@stu.tjau.edu.cn (T.M.); m13091269519@163.com (M.M.); 2203038182@stu.tjau.edu.cn (X.L.); zhangll20@126.com (L.Z.); yiwenguo33@163.com (Y.G.); zerocatlxg@163.com (X.L.); xiangbinding@tjau.edu.cn (X.D.); guohong64@163.com (H.G.)

**Keywords:** *MSTN*, *PSMA6*, skeletal muscle satellite cells, AKT/mTOR, differentiation

## Abstract

*MSTN* has been used as a candidate gene in the genetics, breeding, and improvement of animal breeds. However, the possible mechanism by which the *MSTN* gene regulates muscle development through *PSMA6* is not well understood. Previous methylome and transcriptome sequencing analyses of gluteal muscle tissues from *MSTN*+/−Luxi cattle and wild-type Luxi cattle identified that the *PSMA6* gene exhibited a negative correlation between methylation levels and transcriptional activity. To investigate whether *MSTN* expression regulates *PSMA6* gene expression, we examined the effects of *MSTN* on DNA methyltransferases (DNMT1, DNMT2, DNMT3A, and DNMT3B) and DNA demethylases (TET1, TET2, and TET3). Additionally, chromatin immunoprecipitation (ChIP) assays were performed to detect the binding interaction between *PSMA6* and TET2. In this paper, we first established an MSTN knockdown cellular model to preliminarily validate its regulatory effect on PSMA6 expression. Subsequently, the developmental impact of PSMA6 on bovine skeletal muscle satellite cells was further investigated through both knockdown and overexpression of the PSMA6 gene. Furthermore, we examined changes in the expression of key components of the AKT/mTOR signaling pathway to elucidate the mechanisms underlying the *PSMA6*-mediated regulation of satellite cell development. The results demonstrate that myostatin (*MSTN*) inhibition significantly decreased proteasome 20S subunit alpha-6 (*PSMA6*) gene expression, while increasing demethylase expression, particularly ten-eleven translocation-2 (TET2), which exhibited the most pronounced changes. During the cell proliferation stage, the markers Paired Box 7 (PAX7) and Ki-67 exhibited no significant changes, whereas the *PSMA6* gene was either overexpressed or disrupted. Conversely, *PSMA6* overexpression altered the myogenic differentiation markers, causing the differential regulation of myosin heavy chain (MyHC) and myogenin (MyoG) expression, with MyHC upregulation and concurrent MyoG downregulation. *PSMA6* gene overexpression led to the downregulation of AKT1 and Rac1, as well as the activation of the AKT/mTOR pathway, including key factors such as mTOR, p-mTOR, RPS6, p-RPS6, and RhoA. *PSMA6* interference resulted in the downregulation of p-mTOR and the upregulation of p-RPS6. Gene expression profiling in our study revealed that the myostatin (*MSTN*) knockout model significantly reduced the transcriptional levels of the proteasome α6 subunit (*PSMA6*) (*p* < 0.05), with the regulatory intensity showing a significant negative correlation with *MSTN* expression. This molecular evidence substantiates a negative regulatory axis between *MSTN* and *PSMA6*. Functional experiments demonstrated that *PSMA6* overexpression specifically enhanced myotube formation rates in bovine skeletal muscle satellite cells, whereas siRNA-mediated *PSMA6* knockdown exhibited no significant effects on cellular proliferation, indicating the functional specificity of this gene in myogenic differentiation. Mechanistic investigations further revealed that *PSMA6* activates the canonical AKT/mTOR signaling transduction cascade through the phosphorylation of AKT and its downstream effector mTOR, thereby mediating the expression of myogenic regulatory factors MyoD and myogenin. Collectively, these findings demonstrate that *MSTN* deficiency alleviates the transcriptional repression of *PSMA6*, remodels skeletal muscle differentiation-associated signaling networks, and ultimately drives the directional differentiation of satellite cells toward myofiber specification.

## 1. Introduction

Beef producers must concentrate on raising beef production to fulfill market demand as consumers’ quality of life improves and their desire for meat products rises. The beef industry is constantly improving efficiency and profitability, and the basic development of muscle is very important for the growth and development of beef cattle [1,2]. Growing and developing skeletal muscle involves activating or silencing many genes in a highly complex regulatory network [3,4].

Scientists discovered in 1961 that muscle satellite cells can regenerate repeatedly throughout life but not as a functional unit; instead, they provide the necessary components for damaged cells to repair and rebuild [5]. Satellite cells typically remain quiescent, residing in a specialized niche beneath the basal lamina of muscle fibers, where they await activation signals. Upon activation, these cells undergo proliferation and differentiation into myoblasts, which serve as primary progenitor cells expressing key myogenic regulatory factors. Myoblasts subsequently fuse with existing muscle fibers through a process termed myogenic differentiation. Skeletal muscle, constituting up to 70% of mammalian body mass, represents the largest tissue compartment and provides essential protection for internal organs against mechanical trauma [6,7]. It is worth noting that the growth and development of skeletal muscle directly impacts on the production of beef, as well as other traits closely related to economic benefits, and is therefore considered to be an essential research area in the livestock industry [8]. Studying the role of genes in muscle development helps us uncover how genes regulate the formation of muscles and provides a theoretical basis for treating muscle diseases and promoting muscle growth [9,10].

Myostatin (*MSTN*), also known as growth differentiation factor 8, or GDF-8, is a cytokine that is produced and released by muscle cells. It can inhibit the production of muscle cells and affect their autocrine function. It is a member of the TGF β protein family and is encoded by the *MSTN* gene, which is widely expressed in the genomes of cattle, sheep, pigs, dogs, rats, and even humans [11,12,13,14,15]. As early as 1997, scholars found that mutating *MSTN* genes will lead to double muscle phenotypes in cattle, confirming that *MSTN* is an indispensable part of muscle development and that inhibiting *MSTN* can promote muscle development. This major discovery helps to further the analysis of the *MSTN* self-regulation mechanism and provides new insights into cattle genetics and breeding [16,17]. Moreover, it lays a practical foundation for animal husbandry genetics and breeding to improve the quality of beef production to a large extent. Based on the influence of *MSTN* on animal husbandry genetics and breeding, some scholars have continuously used the optimized Cas9 gene editing system to obtain *MSTN* knockout sheep, knockout pigs, knockout chickens, and knockout mice [18,19,20,21] in order to study the effects of *MSTN* on muscle proliferation and differentiation in vivo, as well as the transcription level of various related factors to lay an experimental foundation for improving the growth of livestock animals and the meat yield and to provide safety data for large-scale use in livestock farms in the future. Numerous investigations have demonstrated that the way in which *MSTN* influences muscle development is complex and that different alterations impact the expression of other defining characteristics. *MSTN* can control the composition of fiber types by controlling the expression of MEF2C and MyoD during myogenesis; when endogenously expressed *MSTN* is knocked down, MyoD and MyoG are upregulated, and Smad3 is downregulated. Furthermore, by eliminating gene methylation, shearing isoform, and directly influencing mitochondrial activity, *MSTN* can also impact muscle growth [22,23,24,25,26].

In our previous study, we performed whole-genome methylation sequencing and transcriptome sequencing on gluteal muscle samples from *MSTN+/−* edited and wild-type Luxi yellow cattle. Integrated analysis identified 11 genes showing decreased promoter methylation and increased transcript levels (negative correlation) after *MSTN* editing (Detailed data are available in the Supplementary Materials section). Through a literature review and through bioinformatics analysis of these 11 genes, *PSMA6* was selected based on two criteria: the highest differential expression ranking and documented evidence supporting its potential involvement in muscle development. This systematic approach led us to prioritize *PSMA6* as the key downstream mediator of *MSTN* regulation.

**Figure 1:**
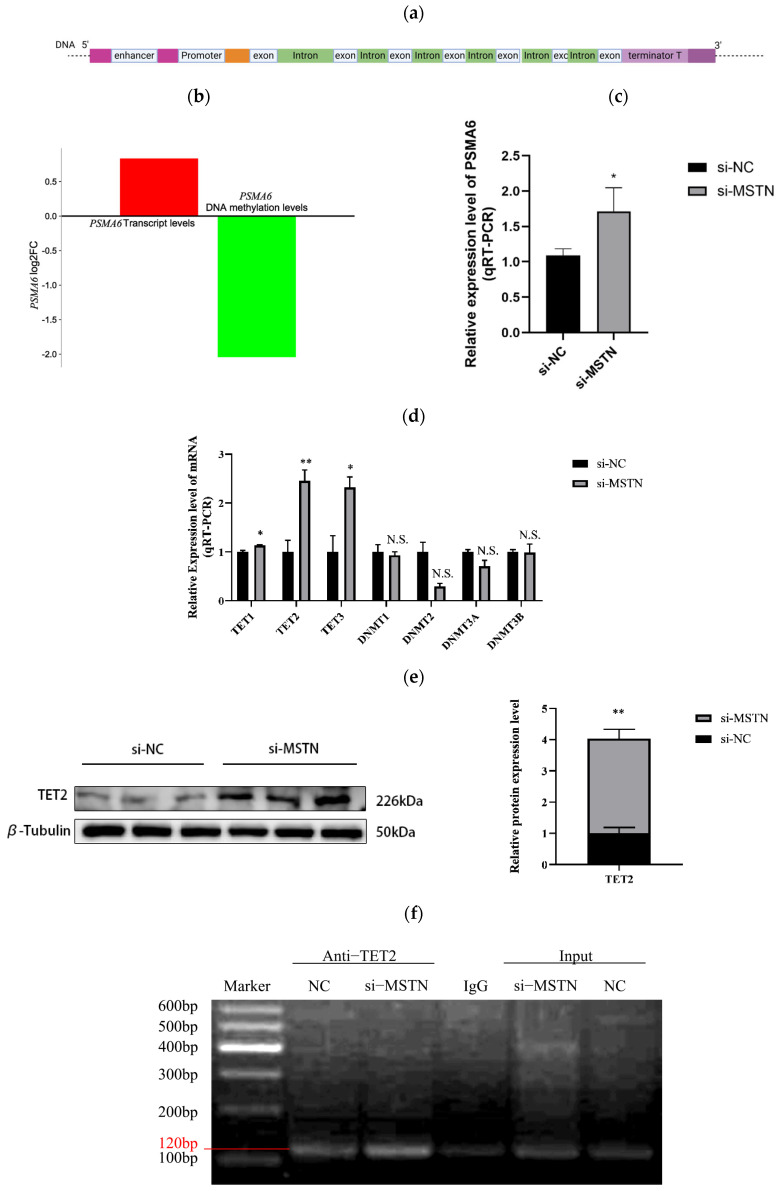
*MSTN* negatively regulates the *PSMA6* gene: (**a**) Schematic representation of the *PSMA6* gene structure (simplified illustration of exon–intron organization and Promoter). Promoter region: Chr12: 55,429,093–55,431,093, GRCh39. (**b**) Fold-difference plot of *PSMA6* gene transcriptome and methylation sequencing data. (**c**) qRT-PCR results of *PSMA6* at mRNA levels after knockdown of *MSTN*. (**d**) qRT−PCR results of mRNA levels for DNMTs and TETs family genes after *MSTN* interference. (**e**) Western blot was used to detect the expression of the TET2 protein. (**f**) ChIP validation of *PSMA6* gene promoter region binding demethylase 2 (TET2). The IgG group served as the negative control, whereas the Input group functioned as the positive control. si−*MSTN* represents *MSTN*−interfered cell samples; si−NC denotes the non-targeting control group (negative control without RNA interference). “**” indicates that the difference is extremely significant (*p* < 0.01), “*” indicates that the difference is significant (*p* < 0.05), and “N.S.” indicates that the difference is not significant (*p* > 0.05).

Proteasome 20S subunit alpha-6 (*PSMA6)* is the α6 subunit of the proteasome, which is present in both the cytoplasm and the nucleus (Figure 1a). In organisms, it directly regulates protein turnover by degrading misfolded proteins or those at the end of their lifecycle. *PSMA6* was selected as the focus of this study due to its role as the catalytic core subunit of the 20S proteasome, which directly regulates ubiquitin-dependent protein degradation, a critical process for modulating key signaling pathways such as AKT/mTOR. Therefore, *PSMA6* was chosen as the candidate gene. The *PSMA6* gene has not been extensively studied before, and through its influence on ubiquitin proteasome function, *PSMA6* further influenced the expression of genes involved in the NF-kB dependent inflammatory pathway. The G2/M phase cell cycle arrest or apoptosis caused by low *PSMA6* expression makes it a promising gene for myocardial infarction research and lung cancer treatment. By upregulating the expression of the transcription factors CCAAT/enhancer binding protein α (*CEBPA*) and peroxisome proliferator activating receptor γ (*PPARG*), as well as important genes linked to lipogenesis, according to a recent study conducted in adipocytes, *PSMA6* supports cell proliferation and speeds up cell division [27,28,29]. Nevertheless, little is known about how *PSMA6* regulates muscle growth and differentiation. Transcriptome sequencing identified *PSMA6* as a key candidate gene. Its mechanistic role in myogenesis warrants further investigation due to its limited characterization in muscle development contexts.

In light of the above, we were prompted to investigate whether *MSTN* influences *PSMA6* expression, which may consequently modulate satellite cell differentiation and proliferation. Subsequently, experimental validations were conducted in bovine skeletal muscle satellite cells, specifically aimed at elucidating the functional involvement of *PSMA6* in mediating *MSTN*’s regulatory effects on these cells. Collectively, these molecular-level discoveries establish a theoretical framework for developing strategies to enhance meat production while providing mechanistic insights into *PSMA6*-mediated regulation during myogenic differentiation.

## 2. Results

### 2.1. Subsection

#### 2.1.1. MSTN Negatively Regulates PSMA6 Gene and TET2 Protein Expression

In preliminary experiments, integrated analysis of methylation sequencing and transcriptome sequencing performed on gluteal muscle tissue from *MSTN*-edited Luxi cattle and their wild-type counterparts revealed a negative correlation between the transcriptional activity of the *PSMA6* gene and the methylation levels (Figure 1b). We employed cell studies to preliminary show that *MSTN* controls the expression of *PSMA6* by controlling the expression of demethylase in order to validate the aforementioned sequencing data (Figure 1c). Using qRT-PCR assays, we investigated the alterations in methyltransferases (DNMTs) and demethylases (TETs) expression at the mRNA level in order to look for variations in the methylation levels of the *PSMA6* gene [30]. The findings demonstrated that whereas the mRNA expression level of methylase remained unchanged, the mRNA expression level of demethylase was markedly elevated. Additionally, the TET2 alteration was the most substantial, and the TET2 protein expression showed the same outcome (Figure 1d,e). We decided to use ChIP experimental technology to pull down the bound DNA fragments with particular antibodies to TET2 in order to further illustrate the binding relationship between the demethylase TET2 and the *PSMA6* promoter region. The band intensity at 120 bp indicates the binding capacity between TET2 demethylase and the *PSMA6* gene. This showed that there was some binding between TET2 and the *PSMA6* gene’s promoter region (Figure 1f). The IgG group served as the negative control, whereas the input group functioned as the positive control. The faint band observed in the IgG control group likely resulted from minor sample carryover during experimental procedures.

#### 2.1.2. PSMA6’s Impact on the Growth of Satellite Cells in Bovine Skeletal Muscle PSMA6 Gene Expression over Time

**Figure 2:**
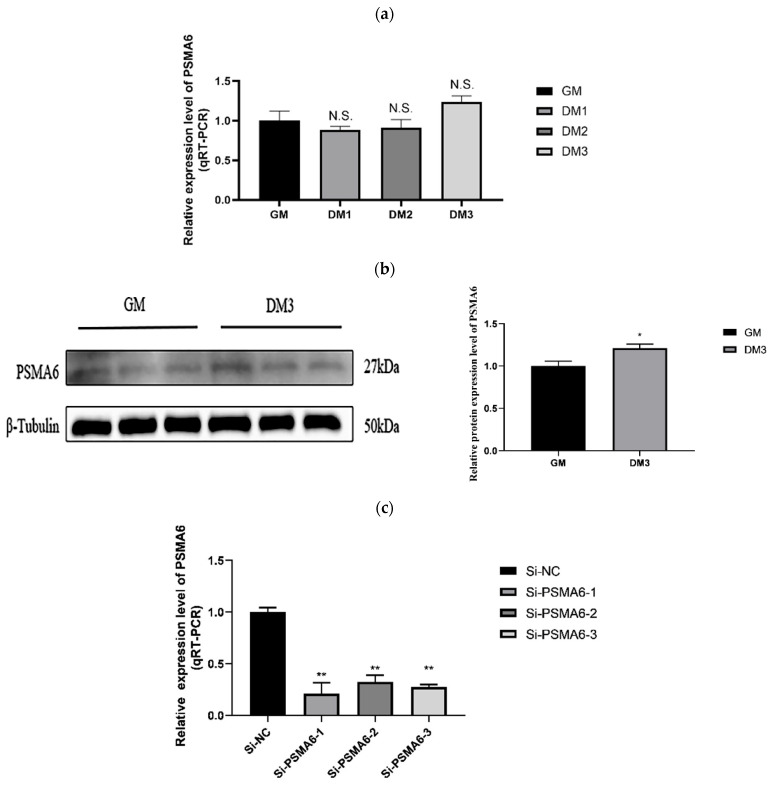
*PSMA6* gene expression level and *PSMA6* inhibition cell model construction: (**a**) qRT-PCR was used to detect the timing expression of *PSMA6* mRNA in bovine skeletal muscle satellite cells during the proliferation and differentiation stages; (**b**) Western blot was used to detect the expression level of *PSMA6* protein and the results of Western blot quantification of *PSMA6* protein; (**c**) qRT-PCR results of mRNA levels of the *PSMA6* gene after three si-RNAs were transfected into bovine skeletal muscle satellite cells for 24 h. “**” indicates that the difference is extremely significant (*p* < 0.01), “*” indicates that the difference is significant (*p* < 0.05), and “N.S.” indicates that the difference is not significant (*p* > 0.05).

We employed PCR to determine the mRNA level of *PSMA6* in bovine muscle cells. We repeated this four times: during proliferation (GM), the first day of differentiation (DM1), the second day (DM2), and the third day (DM3) (Figure 2a). We detected protein expression levels during the cell proliferation phase (GM) and on the third day of differentiation (DM3) using Western blot analysis (Figure 2b). Temporal discrepancies in sampling intervals lead to continued protein accumulation, even after the mRNA levels have peaked. Based on these mechanisms, during day 3 of bovine skeletal muscle satellite cell differentiation, *PSMA6* protein expression reaches its zenith precisely when its corresponding mRNA has undergone degradation, thereby manifesting discordant expression patterns at transcriptional and translational levels. In this experimental system, protein expression levels were used as the primary analytical criterion. The results indicated that *PSMA6* mRNA expression in bovine muscle cells rose over time. No significant difference was found. However, *PSMA6* expression increased at the protein level on day 3. The timing results show that the *PSMA6* gene has a role in muscle cell differentiation. We transfected each of the three designed siRNAs into bovine skeletal muscle satellite cells. For further research, we selected one siRNA with the strongest interference impact (Figure 2c).

##### Regulatory Effects of *PSMA6* on Bovine Skeletal Muscle Satellite Cell Proliferation

**Figure 3:**
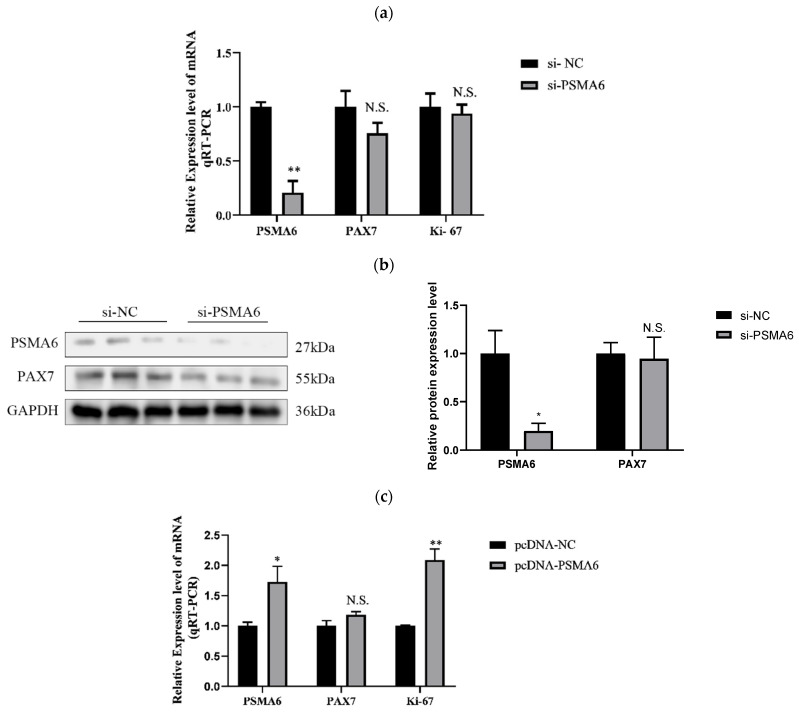

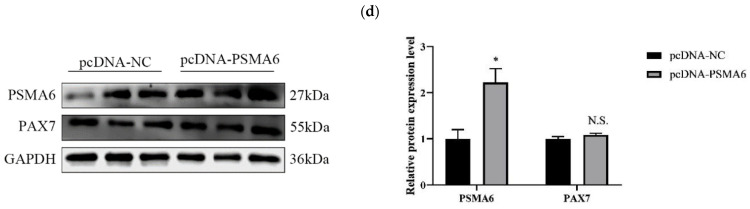
Effect of interference and overexpression of *PSMA6* on the proliferation of bovine skeletal muscle satellite cells: (**a**) alteration of mRNA-level proliferation marker factors following disruption of *PSMA6* gene expression; (**b**) protein-level changes in proliferation marker factors following disruption of *PSMA6* gene expression; (**c**) proliferation marker factor changes at the mRNA level following *PSMA6* gene overexpression; (**d**) following *PSMA6* gene overexpression, the proliferation marker factor’s appearance varies at the protein level. si-*PSMA6* denotes the *PSMA6*-silenced cell sample group (with RNA interference targeting *PSMA6*), while si-NC represents the non-targeting control group (negative control without RNA interference). pcDNA-*PSMA6* indicates the *PSMA6*-overexpressing cell sample group (with ectopic *PSMA6* expression), and pcDNA-NC corresponds to the empty vector control group (without exogenous gene overexpression). “**” indicates that the difference is extremely significant (*p* < 0.01), “*” indicates that the difference is significant (*p* < 0.05), and “N.S.” indicates that the difference is not significant (*p* > 0.05).

To assess the influence of the *PSMA6* expression level on the proliferation period of bovine skeletal muscle satellite cells, we disrupted or overexpressed it by transfection with siRNA and the overexpression vector and tested the effect of the *PSMA6* gene at the mRNA and protein levels by RT-qPCR (Figure 3a) and Western blot (Figure 3b). We discovered that the downregulation and overexpression of *PSMA6* had no effect on the cycle factors implicated in cell proliferation, such as the levels of PAX 7 and Ki-67 protein expression and PAX 7 mRNA levels (Figure 3a,b,d). Only the overexpression of *PSMA6* significantly boosted the mRNA expression of Ki-67 (Figure 3c). According to these findings, *PSMA6* might not be a crucial modulator of satellite cell proliferation in bovine skeletal muscle.

##### *PSMA6* Modulates Differentiation in Bovine Skeletal Muscle Satellite Cells

**Figure 4:**
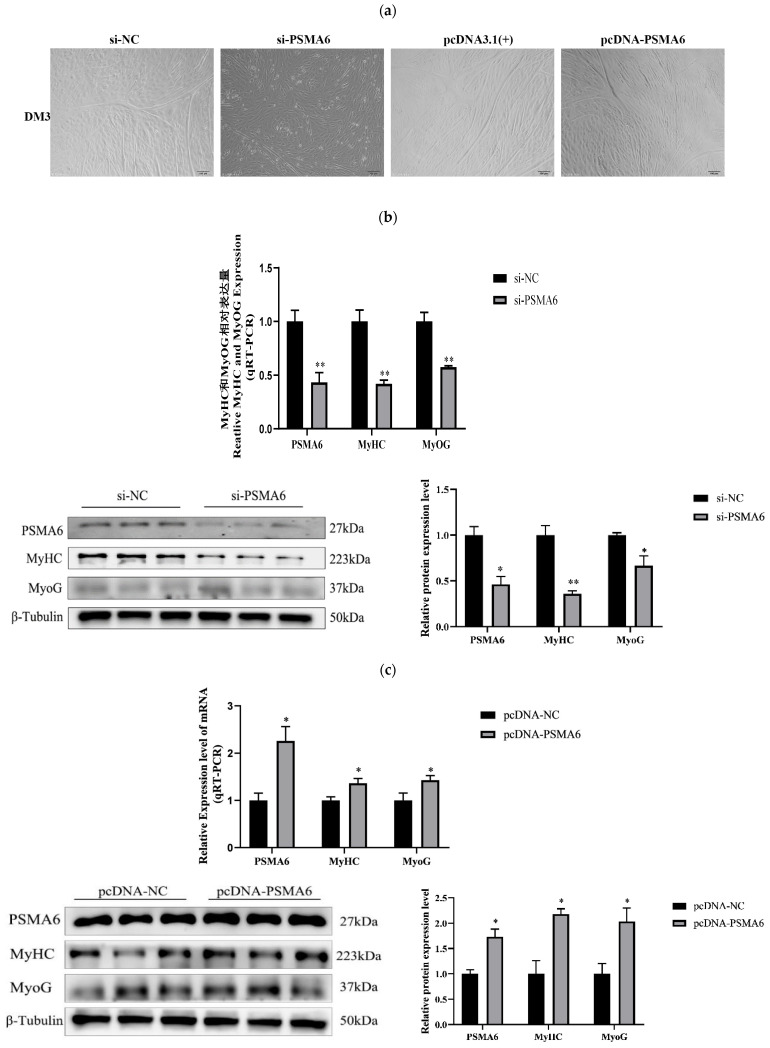
Effect of interference and overexpression of *PSMA6* on the differentiation of bovine skeletal muscle satellite cells: (**a**) plot of cell differentiation development using light microscopy following *PSMA6* gene inhibition and overexpression (100×); (**b**) following *PSMA6* expression interference, the differentiation marker factors MyHc and Myog are expressed at the mRNA and protein levels; (**c**) following *PSMA6* overexpression, the differentiation marker factors MyHc and Myog are expressed at the mRNA and protein levels. si-*PSMA6* denotes the *PSMA6*-silenced cell sample group (with RNA interference targeting *PSMA6*), while si-NC represents the non-targeting control group (negative control without RNA interference). pcDNA-*PSMA6* indicates the *PSMA6*-overexpressing cell sample group (with ectopic *PSMA6* expression), and pcDNA-NC corresponds to the empty vector control group (without exogenous gene overexpression). “**” indicates that the difference is extremely significant (*p* < 0.01), “*” indicates that the difference is significant (*p* < 0.05).

In the meantime, we continue to look into how the *PSMA6* gene influences satellite cell differentiation stage in bovine skeletal muscle. Similarly, we used RT-qPCR and Western blot experimental techniques to examine the effects of interference and overexpression of the *PSMA6* gene on the differentiation stage. First, light microscopy showed that the *PSMA6* gene positively regulates the myotube formation of satellite cells in bovine skeletal muscle (Figure 4a). The findings also demonstrate that following the interruption of *PSMA6* gene expression, differentiation marker factors, including MYHC and MYOG, are markedly downregulated at the mRNA and protein levels (Figure 4b). However, the expression of the aforementioned differentiation marker factors was markedly elevated at both the mRNA and protein levels following *PSMA6* gene overexpression (Figure 4c), which is in line with the light microscopy profile.

In summary, the quantification of proliferation/differentiation markers revealed no significant association between *PSMA6* expression and satellite cell proliferation but demonstrated positive correlation with differentiation capacity in bovine skeletal muscle.

#### 2.1.3. Effects of Interference and Overexpression of PSMA6 on AKT/mTOR Pathway

**Figure 5:**
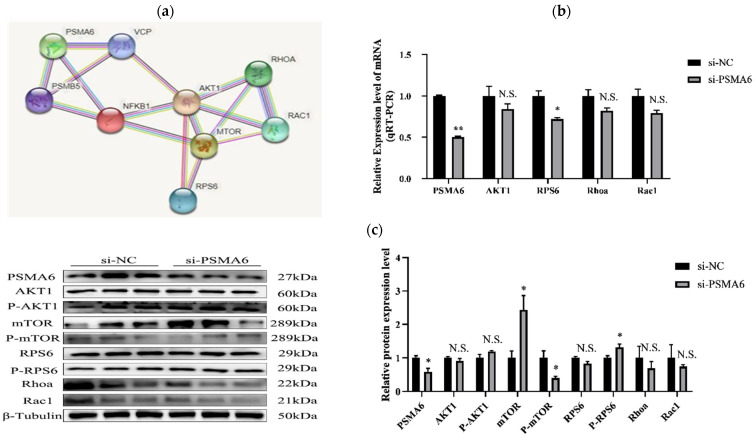

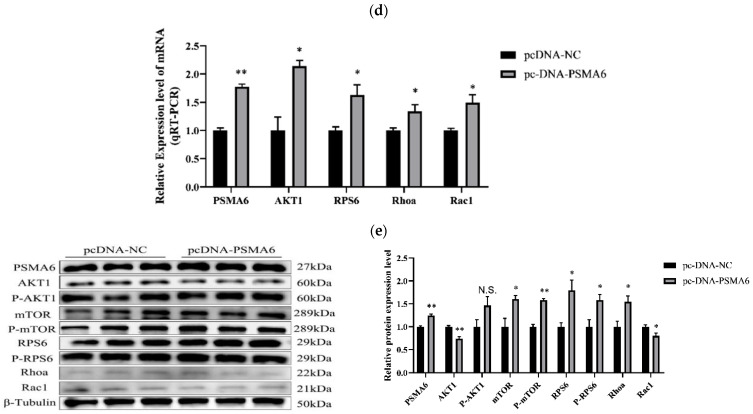
Effects of interference and overexpression of *PSMA6* on the AKT/mTOR pathway: (**a**) STRING predicts the interaction of the *PSMA6* protein with key proteins in the AKT/mTOR pathway; (**b**) detection of key genes in the AKT/mTOR pathway by qRT-PCR at the mRNA transcriptional level after *PSMA6* interference; (**c**) Western blot analysis was performed after *PSMA6* interference to detect the expression of key proteins in the AKT/mTOR pathway; (**d**) key genes in the AKT/mTOR pathway were identified through qRT-PCR analysis of mRNA transcription levels following *PSMA6* overexpression; (**e**) Western blot was utilized to detect the expression of key proteins in the AKT/mTOR pathway following *PSMA6* overexpression. si-*PSMA6* denotes the *PSMA6*-silenced cell sample group (with RNA interference targeting *PSMA6*), while si-NC represents the non-targeting control group (negative control without RNA interference). pcDNA-*PSMA6* indicates the *PSMA6*-overexpressing cell sample group (with ectopic *PSMA6* expression), and pcDNA-NC corresponds to the empty vector control group (without exogenous gene overexpression). “**” indicates that the difference is extremely significant (*p* < 0.01), “*” indicates that the difference is significant (*p* < 0.05), and “N.S.” indicates that the difference is not significant (*p* > 0.05).

STRING network analysis identified *PSMA6* as a potential modulator of bovine myocyte differentiation through AKT/mTOR signaling pathway interactions (Figure 5a). On this premise, we used RT-qPCR and Western blot to analyze changes in the expression of RPS6 pathway-important genes, such as AKT1, RhoA, and Rac 1, as well as the primary protein, such as p-mTOR, P-RPS6, mTOR, AKT1, RPS6, P-AKT1, RhoA, Rac1, according to the results of the *PSMA6* gene interference group.

Only RPS6 revealed reduced mRNA expression, as well as a decreased protein expression of p-mTOR and p-RPS6 (Figure 5b,c). The results of the simultaneous overexpression group indicated that the expression of all four important pathway genes, i.e., AKT 1, RPS6, RhoA, and Rac1, were dramatically elevated at the mRNA level, and the key proteins, i.e., mTOR, p-mTOR, RPS6, an p-RPS6, and RhoA expression, were also dramatically reduced, while the expression of two of these critical proteins, namely, AKT1 and Rac1, were significantly decreased (Figure 5d,e). Determination of the AKT/mTOR signaling pathway’s activation status requires quantitative analysis of the phosphorylated-to-total protein ratio for AKT or mTOR. An elevated ratio corresponds to pathway activation, whereas a reduced ratio reflects pathway suppression. Our experimental findings revealed critical regulatory effects of *PSMA6* on the AKT/mTOR signaling pathway. Specifically, *PSMA6* knockdown induced no significant alterations in AKT phosphorylation levels. However, the phosphorylated-to-total AKT protein ratio was markedly reduced. Concomitantly, both mTOR phosphorylation levels and its phosphorylated-to-total protein ratio exhibited significant reductions, which are collectively indicative of pathway suppression. Conversely, *PSMA6* overexpression resulted in an elevated phosphorylated-to-total AKT ratio despite unchanged absolute phosphorylation levels, alongside significantly enhanced mTOR phosphorylation and an increased phosphorylated-to-total mTOR ratio, which is consistent with pathway activation. The study of how the expression level of *PSMA6* influences the expression of key proteins and key gene mRNA transcript levels in the AKT/mTOR pathway will allow us to better examine the effect of *PSMA6* expression on the AKT/mTOR pathways.

The results of this study demonstrate that when *PSMA6* gene expression occurs in bovine skeletal muscle satellite cells, the mRNA transcription levels for the key genes, i.e., AKT1, RPS6, RhoA, and Rac1, in the AKT/mTOR signaling pathway are markedly elevated when compared to the control group. Notwithstanding the fact that P-AKT1 expression levels do not significantly alter, P-mTOR expression levels are noticeably higher. Also, all major proteins—aside from Rac1—have higher levels of protein expression, including mTOR, RPS6, P-RPS6, and RhoA. In conclusion, the elevated *PSMA6* expression in the satellite cells of the skeletal muscle of cows may encourage the AKT/mTOR pathway to become active. Consequently, the *PSMA6* gene has a favorable regulatory impact on the AKT/mTOR pathway’s activation and inhibition.

## 3. Discussion

*MSTN* is an evolutionarily conserved regulator of myogenesis that maintains myofiber homeostasis through balanced protein turnover. As a promising genetic marker for livestock myogenesis, *MSTN* demonstrates potential for improving production traits. Emerging evidence reveals pleiotropic regulatory functions beyond myogenesis. Metagenomic analyses show distinct microbial signatures between wild-type and *MSTN*-edited sheep models, where *MSTN* ablation enhances growth performance while preserving meat quality parameters [31]. Integrated methylomic and transcriptomic analyses revealed that *MSTN* exerts indirect epigenetic regulation on bovine skeletal muscle satellite cells (bSMCs) through secondary gene networks. This aligns with established mechanisms whereby *MSTN* downregulation activates the *MSTN*–SMAD2/3–TREX1 axis, modulating senescence-associated secretory phenotype (SASP) and delaying cellular aging. Concurrent suppression of TCA cycle/OXPHOS rate-limiting enzymes reduces mitochondrial ATP production, collectively demonstrating *MSTN*’s indirect regulatory role [32,33]. *MSTN* function is tightly regulated through multiple molecular mechanisms. Hormonal modulation of the *MSTN* promoter in mammals alters its activity, while transcription factor NFIX (nuclear factor I X) enhances ovine skeletal myocyte proliferation and differentiation through *MSTN* expression regulation [34,35]. Extensive research has established *MSTN*’s regulatory roles in animal husbandry [21,36,37,38,39,40]. Sequencing analyses revealed that genome-wide expression changes post-*MSTN* knockout, with *PSMA6* showing marked hypomethylation. We first performed *MSTN* knockdown in cell cultures, then measured the expression levels of methylation-modulating enzymes, specifically demethylases (TET1, TET2, TET3) and methyltransferases (DNMT1, DNMT2, DNMT3A, DNMT3B). The results demonstrated that *MSTN* knockdown induced significant upregulation of demethylases at the mRNA level (*p* < 0.05), with TET2 exhibiting a particularly marked increase (*p* < 0.05). In contrast, no statistically significant alterations were observed in DNMT expression (*p* > 0.05). Furthermore, chromatin immunoprecipitation (ChIP) assays were performed to validate the interaction between TET2 and the *PSMA6* gene promoter. Comparative analysis revealed enhanced TET2-*PSMA6* promoter binding affinity under *MSTN*-deficient conditions compared to wild-type controls, providing preliminary evidence that *MSTN* depletion may modulate *PSMA6* methylation status, suggesting that myostatin (*MSTN*) deficiency promotes locus-specific DNA hypomethylation. Coordinated elevation of TET1-3 mRNA/protein levels suggests that active demethylation drives myogenic regulation. These findings align with previous epigenetic studies showing that *MSTN* modulates TET1 via SMAD2/3 signaling to enhance myogenic gene transcription. Our data corroborate that skeletal myogenesis is regulated through TET enzymes and RACK1 demethylation following *MSTN* reduction. While histone acetylation mechanisms (e.g., SFN-mediated MyoD promoter deacetylation) require further investigation, our results expand our understanding of *MSTN*’s epigenetic regulation networks [23,26,41]. The observed TET2 upregulation and *PSMA6* binding correlate with sequencing data, suggesting that TET-mediated 5mC oxidation dynamically regulates developmental gene expression. This extends *MSTN*’s role beyond myostatin encoding, revealing broader epigenetic regulatory significance. *PSMA6* hypomethylation warrants investigation given its proteasomal core function; dysregulation may disrupt proteostasis and drive *MSTN*-deficient hypertrophic phenotypes. While our study establishes an *MSTN*–TET2–*PSMA6* axis, future work should explore its proteasomal and myogenic impacts. Although ChIP data implicate TET2-*PSMA6* in myogenic regulation, potential epigenetic cooperativity necessitates TET2 knockdown/pharmacological inhibition studies to confirm causal relationships in methylation dynamics. We acknowledge the necessity of functional validation for TET2 and have proposed a detailed plan for future studies utilizing CRISPR-Cas9-mediated TET2 knockout models to rigorously test the hypothesis that TET2 is essential for the *MSTN*-mediated regulation of *PSMA6*. The *MSTN* gene’s substantial influence on myogenesis establishes its critical role in livestock breeding programs. As a validated genetic marker, *MSTN* drives innovation in animal biotechnology while motivating mechanistic exploration of its molecular pathways. Genetic marker screening serves as a cornerstone of modern livestock improvement strategies, enabling the systematic elucidation of fundamental biological processes [42,43].

Tissue-specific expression profiling in sheep by Hu et al. revealed ubiquitous *PSMA6* expression, with the highest levels in kidney, liver, and muscle; moderate expression in adipose tissue/spleen/heart; and the lowest pulmonary expression. This differential expression pattern suggests conserved functional significance across species, corroborated by our findings on bovine skeletal myogenesis [44]. Zhao et al.’s multi-stage transcriptomic analysis of Tongcheng and Yorkshire porcine muscle development further supports *PSMA6*’s regulatory role in myogenic networks [45]. Pharmacological *PSMA6* inhibition induces G2/M arrest or apoptosis in cancer models, while its essential role in porcine adipocyte proliferation has been established. Although these findings align with our differentiation data, the absence of proliferative regulation in bovine skeletal muscle satellite cells warrants further investigation into species-specific regulatory mechanisms. We acknowledge that there are limitations in the current experimental design. In subsequent studies, we plan to conduct further experiments to validate the *PSMA6*-DEPTOR interaction and explore its potential crosstalk with other proteasome subunits or upstream regulatory factors. Our study demonstrates the critical regulatory role of the AKT signaling pathway in cellular differentiation processes. Experimental data demonstrate that *PSMA6* facilitates bovine skeletal muscle satellite cell differentiation by activating the AKT/mTOR signaling pathway. This parallels myocardial infarction research where *PSMA6* modulates NF-κB-dependent inflammation via ubiquitination pathways. Notably, NF-κB signaling regulates MyoD expression during skeletal myogenesis, suggesting conserved regulatory networks across tissue types [28,29]. In cancer, *PSMA6* is often upregulated to promote tumor progression by degrading tumor suppressors. Conversely, in skeletal muscle, *PSMA6* promotes the differentiation process of satellite cells in bovine skeletal muscle by activating the AKT/mTOR signaling pathway. Similarly, in adipocytes, *PSMA6* can promote the proliferation of pig adipocytes, a mechanism distinct from its anabolic role in muscle. These discrepancies may arise from differences in interacting partners or tissue-specific post-translational modifications that alter substrate recognition. Our findings identify *PSMA6* as a key developmental regulator in *MSTN*-mediated bovine skeletal myogenesis, providing novel insights into its molecular mechanisms and epigenetic regulatory networks.

It is commonly known that the PI3K/AKT/mTOR signaling pathway is essential for many biological functions, such as glucose metabolism, angiogenesis, apoptosis, and cell division. Recently, research on the AKT/mTOR signaling pathway has mainly focused on these two aspects: cell proliferation and cell apoptosis. The mTOR-mediated Akt signaling can regulate the fusion process of osteoclasts. Additionally, SHMT2 promotes liver regeneration after partial hepatectomy by activating the AKT/mTOR signaling pathway. Fish overexpression of *MSTN*-1 inhibits the activation of the mTOR and AKT/FoxO1 signaling pathways by lowering the phosphorylation level of AKT at the Ser 473 location. Additionally, it has been noted that *MSTN*-1 overexpression enhances FoxO’s dephosphorylation and localization in the cell nucleus [13,27,46,47,48]. The experimental results of these studies demonstrated that AKT/mTOR signaling regulates cell growth and proliferation, indicating that the *PSMA6* gene significantly promotes satellite cell development in dairy cow skeletal muscle. These findings suggest a potential association between *PSMA6* and the AKT/mTOR signaling pathway. We will investigate the transcription and protein expression levels of key pathway genes, including AKT1, p-mTOR, mTOR, RPS6, p-RPS6, RhoA, and Rac1, and analyze the relationship between the *PSMA6* gene and the activation of the AKT/mTOR signaling pathway. While mTOR kinase inhibitor DEPTOR may play a potential role in the *PSMA6*-mediated activation of the AKT/mTOR signaling pathway, our current study lacks direct experimental validation. We have incorporated this aspect into future investigations aimed at systematically identifying *PSMA6*-specific substrates and validating their functional significance in myogenesis. Our comprehensive study will establish a robust theoretical foundation for further exploration.

Our goal was to find out how *MSTN* uses the *PSMA6* gene to seriously control the growth and differentiation of satellite cells in bovine skeletal muscle. The results offer an analytical basis for comprehending how regulatory variables, through *MSTN*, influence skeletal muscle proliferation and differentiation. The regulatory role of *MSTN* in skeletal muscle differentiation may be mediated through modulation of *PSMA6* methylation status, consequently activating the AKT/mTOR signaling pathway. This ultimately influences skeletal muscle differentiation. However, we did not observe an effect of this pathway on skeletal muscle satellite cell proliferation. This reflects that the negative regulation of skeletal muscle differentiation by *MSTN* does not act directly but through intermediate mediators. This finding plays a significant role in elucidating how *MSTN* regulates myogenesis through metabolism.

## 4. Materials and Methods

### 4.1. Cell Isolation and Culture

In this experiment, all cells used were isolated, cryopreserved, and provided by the Key Laboratory of Agricultural Animal Breeding and Healthy Aquaculture of Tianjin. Primary satellite cells were isolated from the gluteus maximus muscle of fetal bovines (gestational age: 5 months).

### 4.2. The Extraction of Total RNA and Quantitative Real-Time PCR

We utilized the EASY spin Plus Organ/Cell RNA Rapid Extraction Kit (Aidlab, Beijing, China) for sterile procedures to isolate total RNA from bovine skeletal muscle satellite cells. The basic steps of the experimental operation are summarized as follows: The lysed cells were added to the DNA-clearing column to remove the DNA in the cell sample. Subsequently, the deproteinization solution, rinsing solution, and eluent solution were added sequentially. The final eluted liquid recovered was the RNA solution, with 3 biological replicates set up in each group. After successfully extracting total RNA from the cells, we used the HiFi Script cDNA synthesis kit provided (Kangwei Biotech, Beijing, China) to synthesize the first strand of cDNA. We then performed qRT-PCR with the 2× All-in-One™ qPCR mix (Genecopoeia, Guangzhou, China). The qPCR protocol is as follows: Step 1—initial denaturation at 95 °C for 60 s (1 cycle); Step 2—denaturation at 95 °C for 10 s; Step 3—annealing at 60 °C for 20 s; Step 4—extension at 72 °C for 15 s (Steps 2–4 are repeated for 40 cycles). Dissociation curve analysis: gradual heating from 72 °C to 95 °C with a ramp rate of 0.5 °C every 6 s. GAPDH mRNA was used as an internal reference control. The relative expression levels of the target genes were calculated using the 2^−ΔΔCt^. Detailed information on the relevant primers is given in Table 1.

### 4.3. Overexpression Vectors and siRNA Synthesis

The *PSMA6* gene was subcloned into the BamHI and EcoRI sites of the pcDNA3.1(+) plasmid to construct the recombinant plasmid pcDNA-*PSMA6*. The siRNA and siNC used for interference experiments were designed and synthesized by Guangzhou RiboBio Technology. The siRNA screening experiment for *MSTN* had previously been completed and verified. Subsequently, these siRNAs were transfected into bovine skeletal muscle satellite cells to evaluate the interference efficiency of the three siRNAs. The most effective siRNA was selected from the three for use in subsequent interference experiments. The specific sequences of the siRNAs are detailed in Table 2.

### 4.4. Plasmid In Vitro Transformation

The successful plasmid pcDNA-*PSMA6* was transformed into DH5α competent cells and subjected to heat shock; then, 500 μL of LB liquid medium, without ampicillin, was added. The mixture was shaken at 37 °C for 60 min, and the supernatant was subsequently discarded. The sample was evenly spread on LB solid Petri dishes containing ampicillin, placed upright at 37 °C for 1 h, and then inverted overnight. After overnight incubation, PCR verification was conducted, followed by Sanger sequencing. The sequencing results were analyzed to identify the bacteria and extract the plasmid.

### 4.5. Cell Transfection

Upon observing that the density of bovine skeletal muscle satellite cells reached 60%, we transfected the cells with siRNA and plasmids using Lipofectamine 3000. After the transfection procedure, the cells were placed in an incubator at 37 °C with 5% CO_2_ for cultivation. Subsequently, we collected the cells at two post-transfection time points: 24 h and 72 h.

### 4.6. Extraction of Protein & Western Blot Analysis

We collected the bovine skeletal muscle satellite cells for the required period, discarded the medium to wash the cells, added 200 μL of RIPA lysate (Solarbio, Beijing, China) containing PMSF to each well of the six-well plate, placed them on ice for 10 min, then collected the cells, centrifuge at 4 °C, 12,000× *g* for 10 min, and stored the supernatant at −80 °C for protein samples. We performed a quantitative analysis of the collected protein samples according to the instructions of the BCA Protein Assay Kit (ComWin Biotech, Beijing, China). After determining and calculating the concentration of the protein samples, we separated the protein molecules on a 10% SDS-PAGE gel. We transferred the separated proteins from the gel to a PVDF membrane and blocked the PVDF membrane with TBST buffer containing 5% milk powder overnight at 4 °C. The next day, we incubated the PVDF membrane containing the protein samples with the primary antibody at 4 °C for one day, then incubated the PVDF membrane with the secondary antibody on a shaker for one hour to detect the exposure. Finally, the grey values of the exposure results were analyzed using ImageJ software 1.8.0.

### 4.7. CHIP

Bovine skeletal muscle satellite cells with knockdown *MSTN* were collected and sonicated on the third day of differentiation. Subsequently, the CHIP Assay Kit (Beyotime Biotechnology, Shanghai, China) instructions were followed, and the bound DNA fragments were pulled down using specific antibodies. All antibody information is shown in Table 3. Due to technical limitations in the initial ChIP assays, qPCR-based measurements were not feasible. At the time of these experiments, our laboratory relied on qualitative gel electrophoresis for ChIP analysis.

### 4.8. Statistical Analysis

Three biological replicates were run for both the experimental and control groups, and data are presented as “mean ± standard error”. Quantitative analysis of Western blot bands was performed using image analysis software. β-tubulin was used as an internal reference protein during Western blot analysis. In addition, GAPDH served as a reference gene during real-time PCR analysis, using sing 2^−∆∆Ct^ to calculate relative gene expression levels. Significance analysis of differences was performed using the *t*-test. Statistically, “**” indicates highly significant differences (*p* < 0.01), “*” indicates significant differences (*p* < 0.05), and “N.S.” indicates non-significant differences (*p* > 0.05).

## 5. Conclusions

The *MSTN* gene negatively regulates the expression level of the *PSMA6* gene, thereby modulating the regulatory effects of the AKT/mTOR signaling pathway, which ultimately influences the bovine myogenic differentiation process. These findings provide novel scientific insights into the mechanistic role of *MSTN* in muscle development.

## Figures and Tables

**Table 1:** All relevant primers of qRT-PCR.

Genes	Primer Sequence (5′-3′)		Product Length (bp)
*PSMA6*	F	GCCCGAGATTGTGCTTGGAA	149
	R	GATGTAAGGCCACCCTGGTT	
MyHC	F	TGCTCATCTCACCAAGTTCC	105
	R	CACTCTTCACTCTCATGGACC	
MyoG	F	CAAATCCACTCCCTGAAA	140
	R	GCATAGGAAGAGATGAACA	
PAX7	F	AGCCAGAGTTTCAACGGGAG	93
	R	GTCGCCAACAGACAACACAC	
Ki-67	F	GAGGTGGCTCAGGTTCGTC	97
	R	AAAGGGTTGGTGGTAAGTGGC	
GAPDH	F	TGTTGTGGATCTGACCTGCC	135
	R	AAGTCGCAGGAGACAACCTG	
*PSMA6*-promoter	F	ATGGCAGGTCAAACCAAAGG	120
	R	ATGGCACGGTCACTGGAAAG	
Rac1	F	ACCCGCAGACAGATGTATTCT	121
	R	AGGATGATGGGTGTGTTGGG	
RhoA	F	TCTTCGAAACGACGAGCACA	100
	R	AGCACCAATCCTGTTTGCCA	
RPS6	F	ATGTTGTGCGAAAGCCCCTA	91
	R	TGCAGAACTCGTGGAGTCAC	
TET1	F	TATCAAAACCAGGTGGCGCT	160
	R	GTTTTATTTCCACTGTGCTCCCA	
TET2	F	GAAAGGAGACCCGACTGCAA	215
	R	TGAATGAATTCAGCAGCTCTGTC	
TET3	F	GGACTCTGCCTTCTGGTGAC	187
	R	GAGGAGAGTTGTGTGAGGGC	
DNMT1	F	TATCGGCTGTTCGGCAACAT	198
	R	TCTGGTGGCAGAAACATGGG	
DNMT2	F	CAGCGATCTCTCTGTGCGAA	388
	R	TCCAAGTAGACGGTAACGCTG	
DNMT3A	F	TTGTCTTGCGTCTCCTTCCC	111
	R	GGAGGAACTGCACTGTCGAA	
DNMT3B	F	GACAAGCACGCCAACAGAAG	188
	R	CTGGAGACCTCCCTCTTGGA	

**Table 2:** All sequences of the siRNAs.

Fragment Name	Sequence (5′-3′)
si-bta-*PSMA6*_001	GAAGAAAGTACCTGACAAA
si-bta-*PSMA6*_002	CCTCTTGGTTGTTGTATGA
si-bta-*PSMA6*_003	GCAGCAGGAGTTAAACAAA

**Table 3:** The antibody information.

Antibody Name	Manufacturer	Catalog Number	Host	Final Concentration
TET2	Abmart (Shanghai, China)	PS04133	Rabbit	1:1000
*PSMA6*	Abmart (Shanghai, China)	TD6911	Rabbit	1:1000
MyoG	DSHB (America)	F5D	Mouse	1:100
MyHC	DSHB (America)	MF20	Mouse	0.5 μg/mL
GAPDH	Zhongshan Golden Bridge (Beijing, China)	TA-08	Mouse	1:1000
AKT1	Abmart (Shanghai, China)	T55561	Rabbit	1:1000
p-AKT1	Abmart (Shanghai, China)	T55885	Rabbit	1:1000
RPS6	Sangon Biotech (Shanghai, China)	D291353	Rabbit	1:1000
P-RPS6	Abmart (Shanghai, China)	TA7331	Rabbit	1:1000
mTOR	Abmart (Shanghai, China)	TA6308	Rabbit	1:1000
P-mTOR	Abmart (Shanghai, China)	S2448	Rabbit	1:1000
Rac1	NewEast (America)	26005	Mouse	1:1000
RhoA	NewEast (America)	26007	Mouse	1:1000

## Data Availability

Data are contained within the article and Supplementary Materials.

## Supplementary Materials

The following supporting information can be downloaded at https://www.mdpi.com/article/10.3390/ijms26114963/s1.

## Abbreviations

The following abbreviations are used in this manuscript:

*MSTN*	Myostatin
*PSMA6*	Proteasome 20S subunit alpha-6
TET1	Tet methylcytosine dioxygenase-1
TET2	Tet methylcytosine dioxygenase-2
TET3	Tet methylcytosine dioxygenase-3
DNMT1	DNA methyltransferase-1
DNMT2	DNA methyltransferase-2
DNMT3	DNA methyltransferase-3
IgG	Immunoglobulin G
CHIP	Chromatin immunoprecipitation assay
AKT/mTOR	Protein kinase B/mammalian target of rapamycin
MyoG	Myogenin
MyHC	Myosin heavy chain
GAPDH	Glyceraldehyde-3-phosphate dehydrogenase
PAX7	Paired Box 7
Ki-67	More than a proliferation marker
AKT1	AKT serine/threonine kinase 1 gene
RPS6	Ribosomal protein S6
RhoA	Ras homolog family member A
Rac1	Ras-related C3 botulinum toxin substrate 1
mTOR	Mammalian target of rapamycin
