# Supplementary material for: MSTN Regulates Bovine Skeletal Muscle Satellite Cell Differentiation via PSMA6-Mediated AKT Signaling Pathway
Source: Int J Mol Sci. 2025 May 22;26(11):4963. doi: 10.3390/ijms26114963 (PMC12154411; doi:10.3390/ijms26114963)
Supplement: Supplementary file 1 [file ijms-26-04963-s001.zip › ijms-3614908-supplementary/3.The integrated analysis data of whole-genome bisulfite sequencing (WGBS) and transcriptomics.docx]

**Whole-genome DNA methylation sequencing study of MSTN+/- gene-edited Luxi yellow cattle**

In the initial phase of the project, we collected gluteal muscle tissues from MSTN+/- edited Luxi yellow cattle and wild-type (WT) counterparts to perform whole-genome bisulfite sequencing (WGBS). Integrated WGBS and bioinformatics analyses identified 3,749 differentially methylated genes (DMGs). For promoter regions, genes exhibiting a fold change ≥2.00 or ≤0.50 in DNA methylation levels (P ≤ 0.05) were identified as significant differentially methylated genes (DMGs), with 22 hypermethylated and 17 hypomethylated genes (Figure 1B and 1C). Hierarchical clustering analysis of all samples and genes demonstrated high correlation and homogeneity (Figure 1D). Furthermore, KEGG and GO analyses were conducted for the 369 promoter-associated DMGs (Figure 1E and F). KEGG pathway enrichment revealed significant associations with osteoclast differentiation, toxoplasmosis, AMPK signaling pathway, and estrogen signaling. GO analysis indicated predominant enrichment in biological processes such as arginine import into the cell, arginine transmembrane transport, and ornithine transport.

C

B

A

D

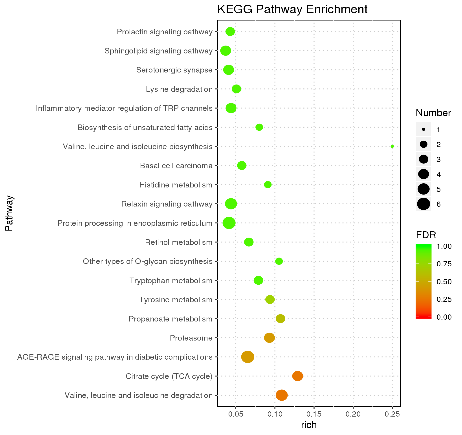

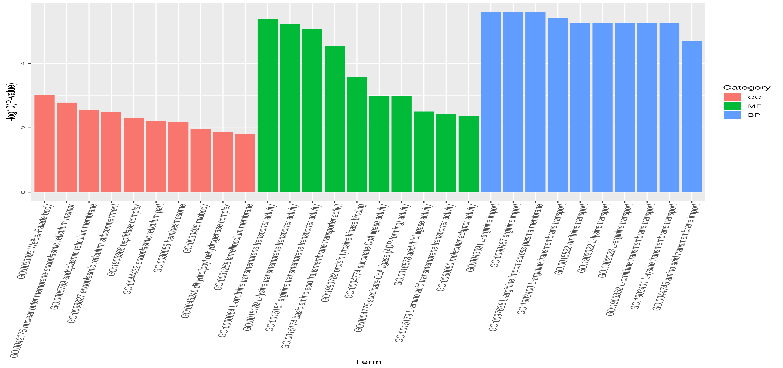


E

F

**Figure 1 MSTN^+/-^ gene edited methylation omics analysis of Luxi yellow cattle and wild-type cattle.**

1. Proportion of gene body and promoter regions identified by WGBS sequencing. B. Statistics of the number of screened differential methylated genes. C. Promoter regional volcano map. D. Clustering heat map of gene expression levels in promoter regions. E. Bubble chart of DNA methylation modification gene KEGG enrichment analysis in gene promoter region. F. Histogram of GO enrichment analysis of DNA methylation-modified genes in the promoter

**Integrated Analysis of Methylomics and Transcriptomics for Identification of Key Differentially Expressed Genes Affecting Muscle Development**

Generally, DNA methylation modifications exhibit a negative correlation with transcriptional expression. To screen for DNA methylation-modified genes that significantly affect muscle development following MSTN expression inhibition, we performed an integrated analysis of methylomics and transcriptomics data from MSTN+/- gene-edited Luxi yellow cattle and wild-type (WT) cattle. Results showed that in the gene body region, 40 genes with a negative correlation to mRNA expression levels were identified from 136 differentially methylated upregulated genes, and 17 such genes were identified from 66 downregulated genes (Fig. 2-A and B). In the promoter region, 4 genes negatively correlated with the mRNA expression level were screened from 22 differentially methylated upregulated genes, and 11 genes negatively correlated with the mRNA expression level were screened from 17 downregulated genes (Figure 2-A and C). Through the above integrated analysis, key differentially expressed genes that may affect muscle development were screened out, providing important targets for further research on the mechanism of MSTN in the follow-up.

B

C

A

**Figure 2 Combining methylomics and transcriptomics analysis to identify the key differentially. expressed genes that affect muscle development**

1. Statistics of DNA methylation differential genes. B. The differential gene statistics of DNA methylation level and mRNA level in gene body region were negatively correlated. C. Promoter region DNA methylation level and mRNA level negative correlation difference gene statistics.
