# Supplementary figures and images for: MSTN Regulates Bovine Skeletal Muscle Satellite Cell Differentiation via PSMA6-Mediated AKT Signaling Pathway
Source: Int J Mol Sci. 2025 May 22;26(11):4963. doi: 10.3390/ijms26114963 (PMC12154411; doi:10.3390/ijms26114963)

**Fig. 2.(e)**

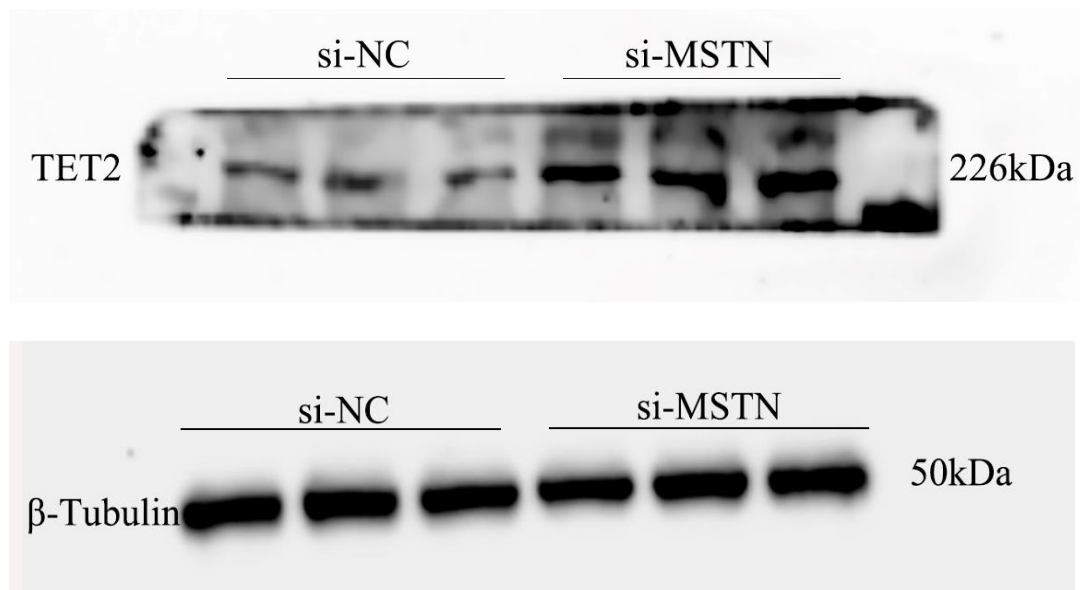

**Fig. 3.(b)**

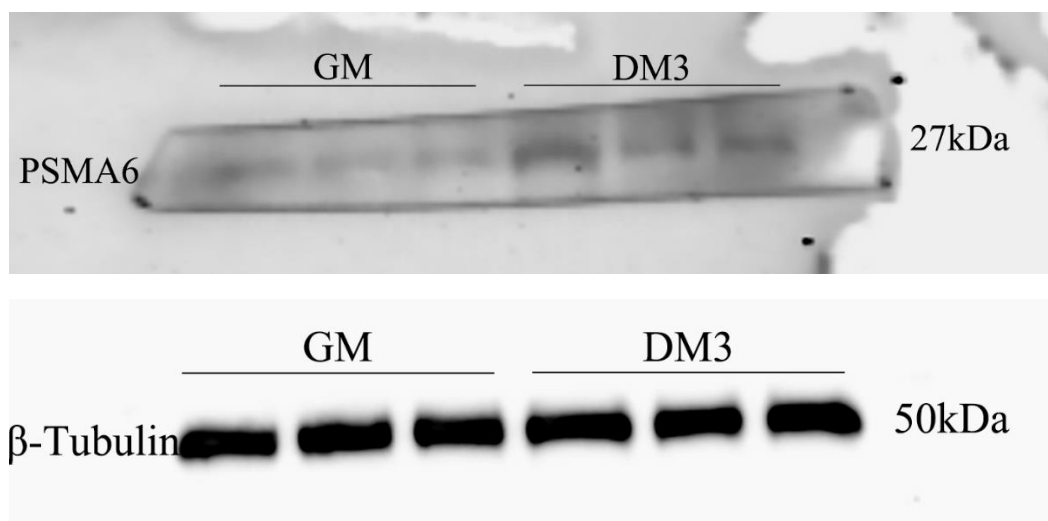

**Fig. 4.(b)**

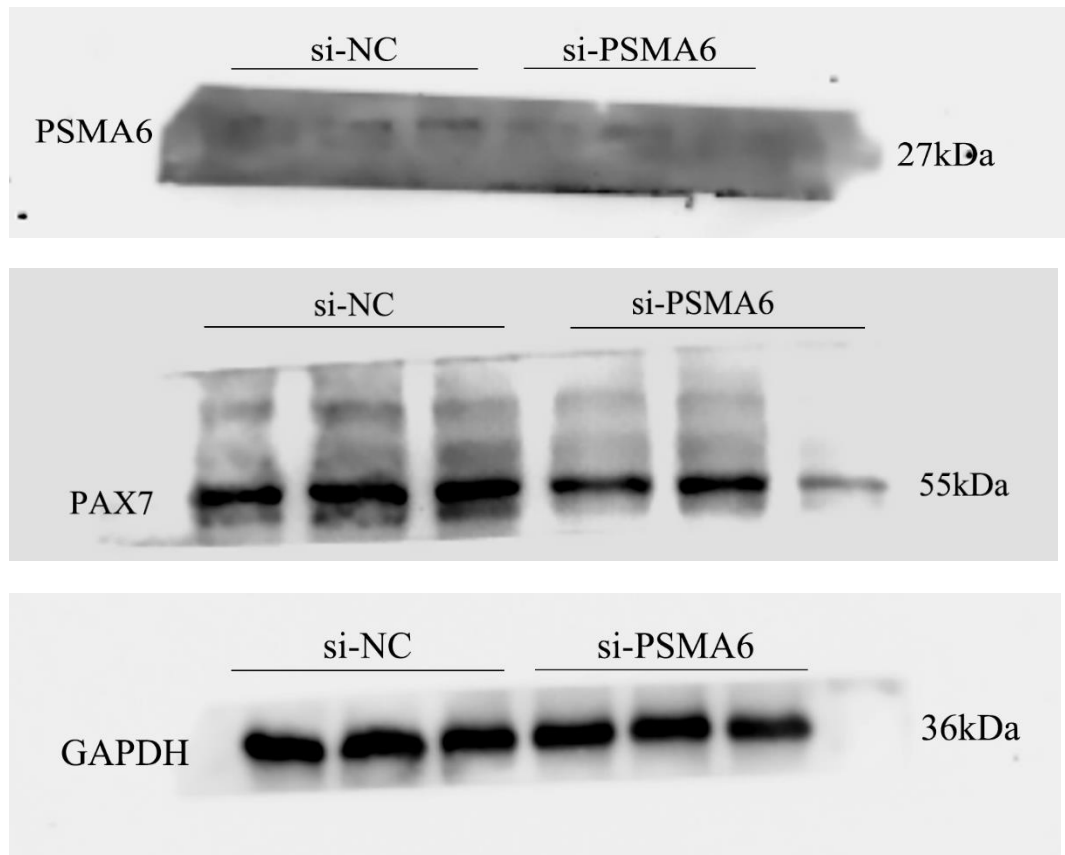

**Fig. 4.(d)**

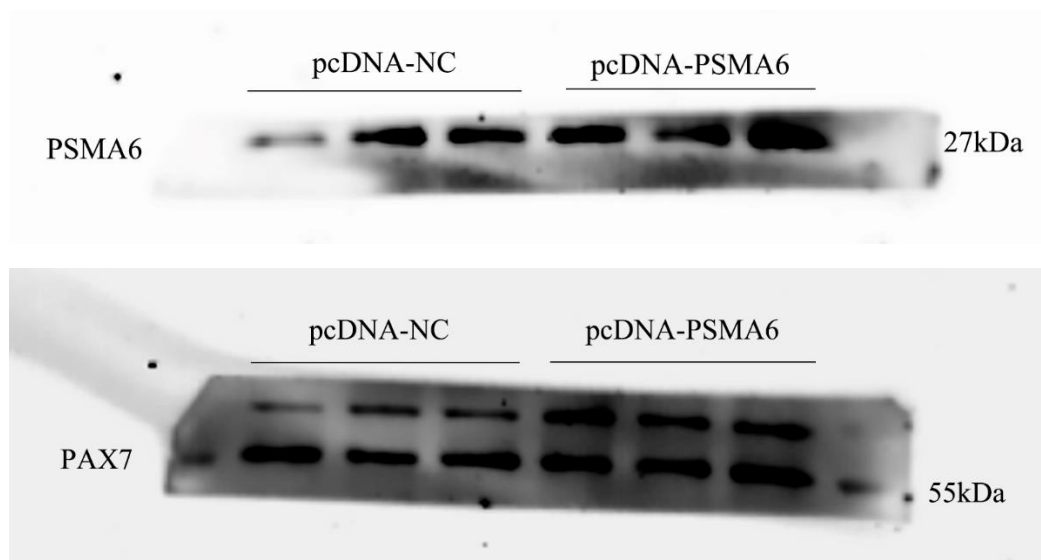

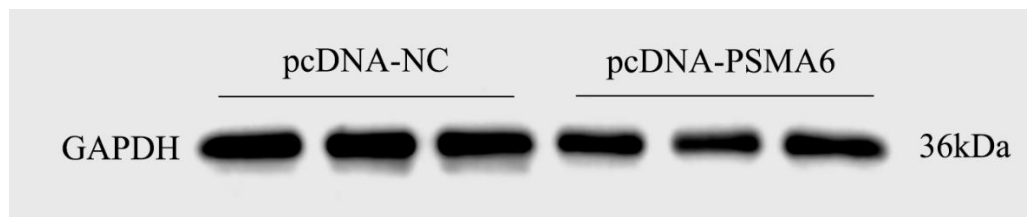

**Fig. 5.(b)**

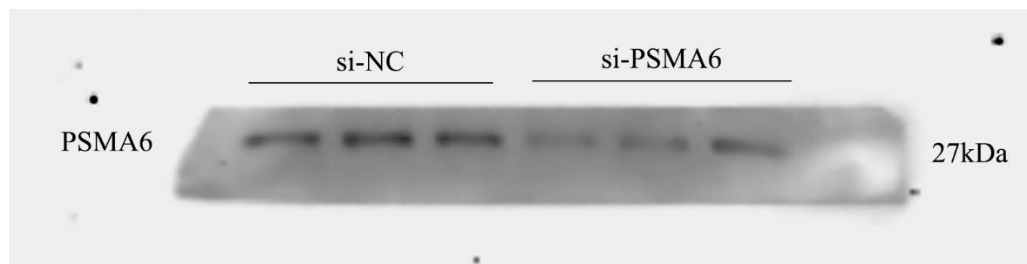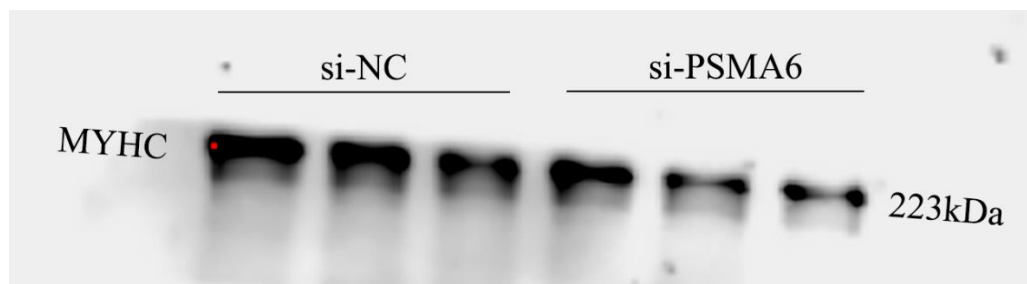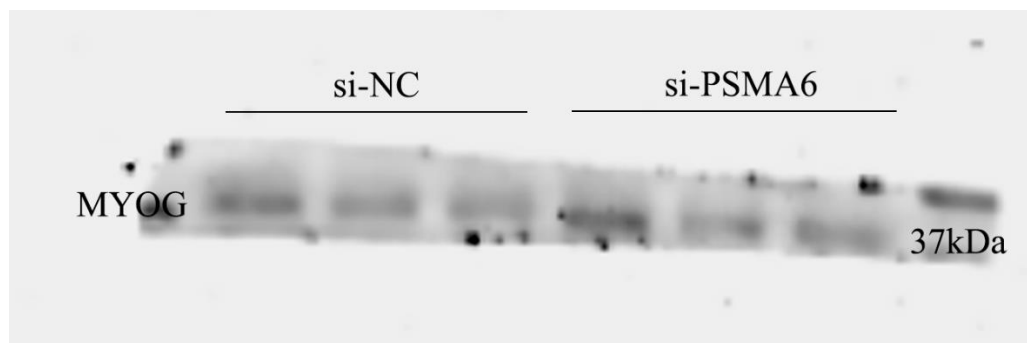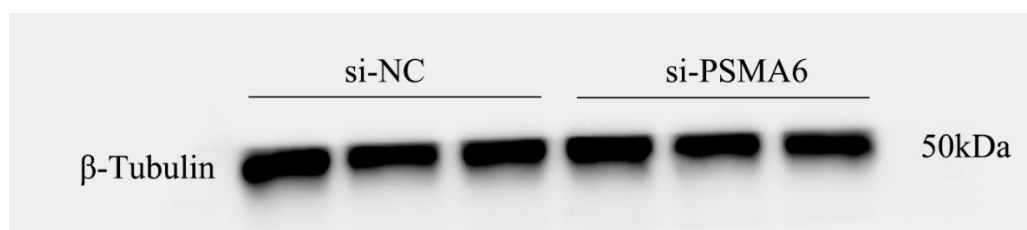

**Fig.5.(c)**

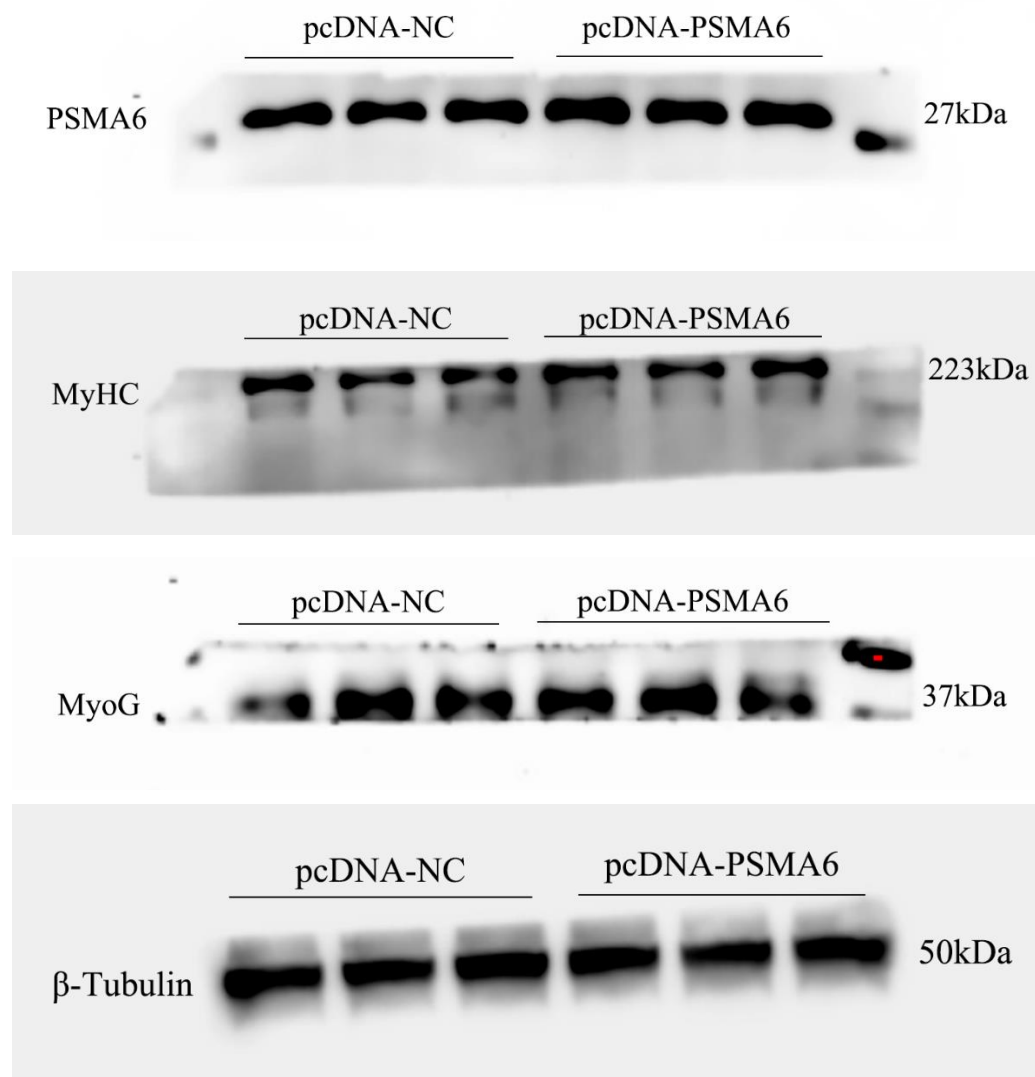

**Fig. 6.(c)**

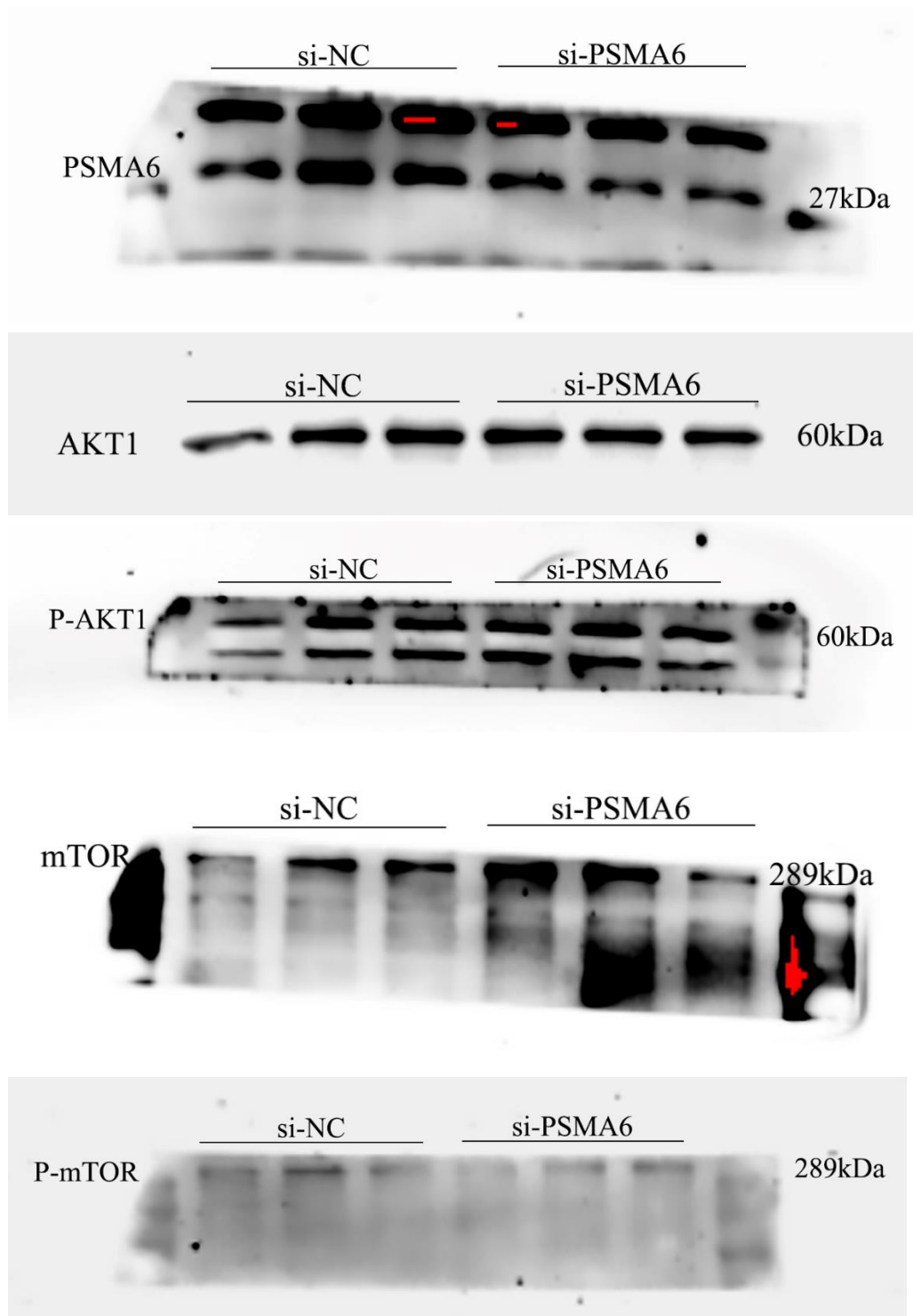

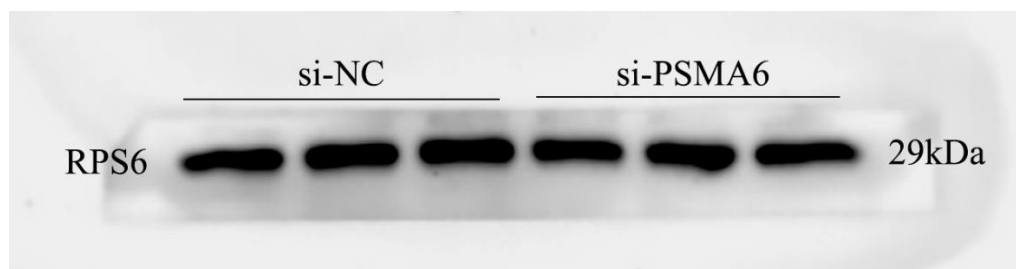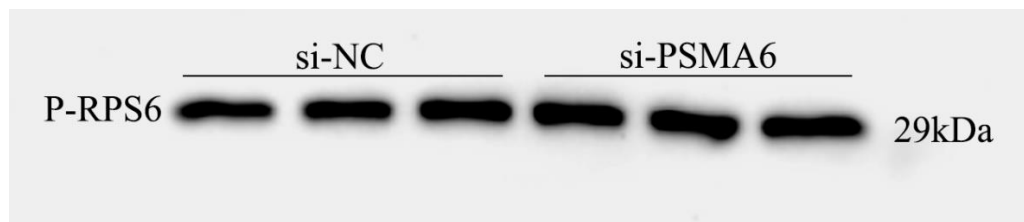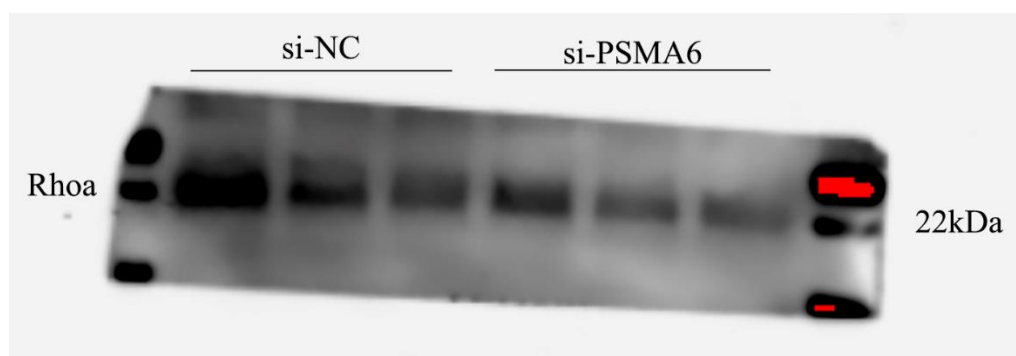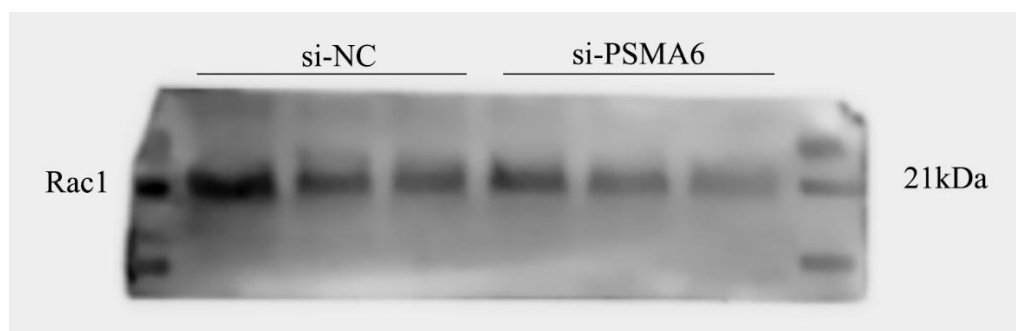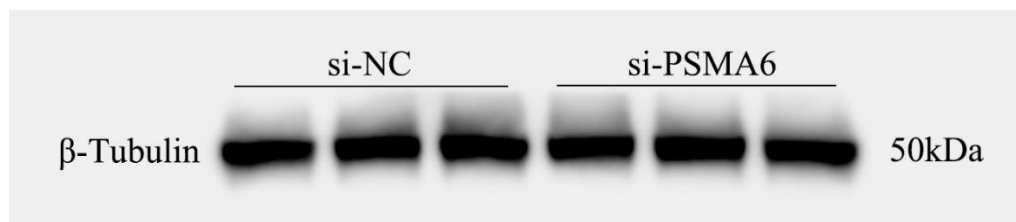

**Fig. 6.(e)**

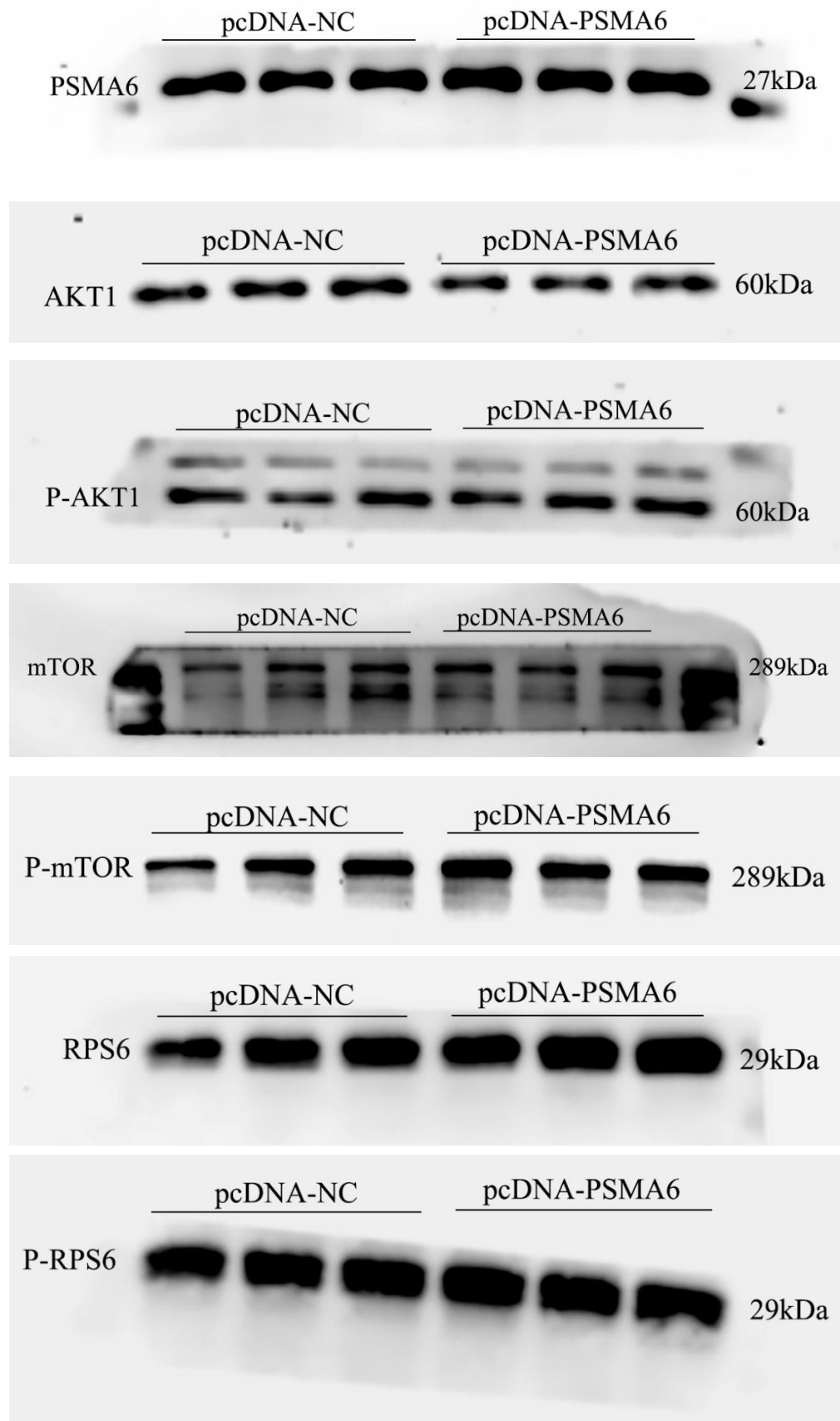

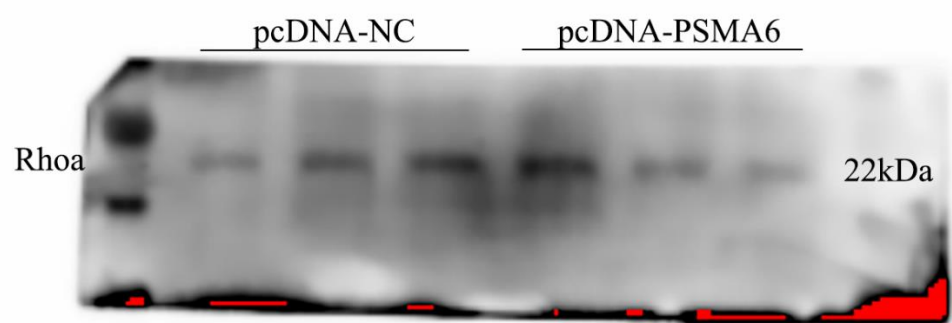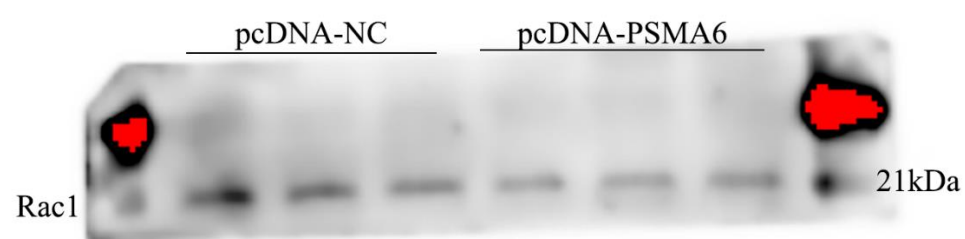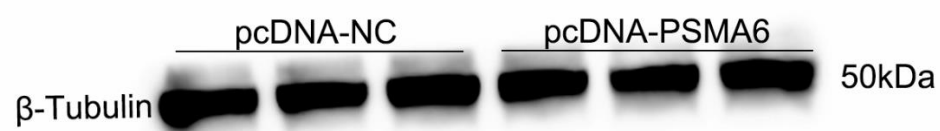

Supplement: Supplementary file 1 [file ijms-26-04963-s001.zip › ijms-3614908-supplementary/blots gels microscopy data.pdf]
