# Supplementary material for: MSTN Regulates Bovine Skeletal Muscle Satellite Cell Differentiation via PSMA6-Mediated AKT Signaling Pathway
Source: Int J Mol Sci. 2025 May 22;26(11):4963. doi: 10.3390/ijms26114963 (PMC12154411; doi:10.3390/ijms26114963)
Supplement: Supplementary file 1 [file ijms-26-04963-s001.zip › ijms-3614908-supplementary/1.Explanation to the reviewers.docx]

Dear Professor,

Regarding the epigenetic modifications discussed in our manuscript (particularly DNA methylation), our primary objective is to demonstrate MSTN's regulatory effect on PSMA6 gene expression. However, as you appropriately noted, our current cellular experimental evidence remains insufficient to establish a definitive causal relationship that MSTN regulates PSMA6 expression through methylation. In accordance with rigorous scientific reporting standards, we have therefore conscientiously refrained from employing methylation-specific terminology in either the title or concluding statements. Instead, we have strategically focused the narrative on our empirically validated findings that the MSTN-mediated regulation of PSMA6 expression. This approach ensures full alignment with our experimental data while maintaining appropriate scientific caution regarding unverified molecular mechanisms (this aspect is fully supported by experimental evidence).

The initial identification of PSMA6 as a candidate gene was derived from integrated analysis of whole-genome methylation sequencing and transcriptome sequencing data from MSTN-deficient bovine muscle tissues (these sequencing datasets are available for review). In cellular experiments, we observed that PSMA6 expression levels significantly decreased (P < 0.05) following MSTN knockdown, confirming MSTN's regulatory effect on PSMA6. Additionally, we analyzed DNA methyltransferases (DNMT1, DNMT2, DNMT3A, DNMT3B) and found no significant changes after MSTN interference. However, demethylases (TET1, TET2, TET3) showed significant upregulation, leading us to hypothesize that MSTN knockdown may enhance genome-wide demethylation activity. To further investigate this mechanism, we conducted Chromatin Immunoprecipitation (ChIP) assays to examine whether MSTN knockdown affects the binding of demethylase TET2 to the PSMA6 gene. The results demonstrated stronger TET2-PSMA6 binding in the MSTN-interfered group compared to the control group, providing mechanistic evidence supporting MSTN's regulatory role on PSMA6. Based on these collective findings, we conclude that MSTN indeed exerts a regulatory effect on PSMA6 gene expression.

Sincerely thank you for your valuable suggestion, which is indeed a highly meaningful and rigorous proposal that has greatly benefited our research. We would like to inquire whether focusing on the perspective of MSTN regulating PSMA6 expression without emphasizing methylation as the central focus would align with the journal's publication requirements. We would be most grateful for your professional guidance on this matter, Professor.

Best regards,

Dr. Hu
